# Teledermatology viewpoint: Sudden onset of a widespread rash

**DOI:** 10.1016/j.jdcr.2024.10.034

**Published:** 2024-11-29

**Authors:** Alexis R. Bernat, Robert T. Brodell, Lindsey B. Dolohanty

**Affiliations:** a MS-2, School of Medicine, Case Western Reserve University, Cleveland, Ohio; b Department of Nutrition, School of Medicine, Case Western Reserve University, Cleveland, Ohio; c Department of Dermatology, School of Medicine, University of Mississippi Medical Center, Jackson, Mississippi; d Department of Pathology, School of Medicine, University of Mississippi Medical Center, Jackson, Mississippi; e Department of Dermatology, G.V. (Sonny) Montgomery Veterans Administration Hospital, Jackson, Mississippi; f Dermatology Section, Department of Dermatology, Northeast Ohio Medical University, Rootstown, Ohio

**Keywords:** anemia, erythema infectiosum, fetal, Fifth disease, parvovirus B19, pruritus, teledermatology, viral exanthem

## Introduction

Fifth disease, or erythema infectiosum (EI), is a childhood exanthem that is benign in nature and commonly occurs in school-age children in the early spring and late winter months. The viremia is a result of a parvovirus B19, nonenveloped single-stranded DNA virus. It is highly infectious and spreads in the air through respiratory droplets.^1^ Interestingly, by the time the rash appears, children are no longer infectious.^2^^,^^3^ Patients usually exhibit a nonspecific prodrome or are entirely asymptomatic before the classic skin findings of “slapped-cheeks” and a pruritic, erythematous net-like rash arise.^4^ As the rash fades, the reticular or lace-like pattern may become confluent. Although the disease is self-limited, exercise leading to overheating can lead to reappearance of the erythema, but usually not pruritus, in the areas where rash occurred for weeks to months.^5^ Pramoxine-containing moisturizers, low-potency topical corticosteroids, and oral antihistamines may be used to control any associated pruritus.

EI requires consideration of certain special populations. In adolescents with sickle cell disease, it is associated with red cell aplasia—otherwise known as aplastic crisis. Complications such as acute chest syndrome, splenic sequestration crisis, nephrotic syndrome, stroke, and acute severe pain occur after the viremia phase of EI.^1^ EI can also cause harmful effects to both pregnant mothers and the fetus. Risk of adverse fetal outcomes has been associated with EI, including the development of fetal anemia, fetal hydrops, and fetal death.^6^ No specific treatment is available; however, counseling of at-risk mothers and monitoring of confirmed fetal infections throughout pregnancy will likely decrease fetal mortality.^6^ Here, we present a case that uniquely uses teledermatology as a way to identify and diagnose EI.

## Case report

**Fig 1:**
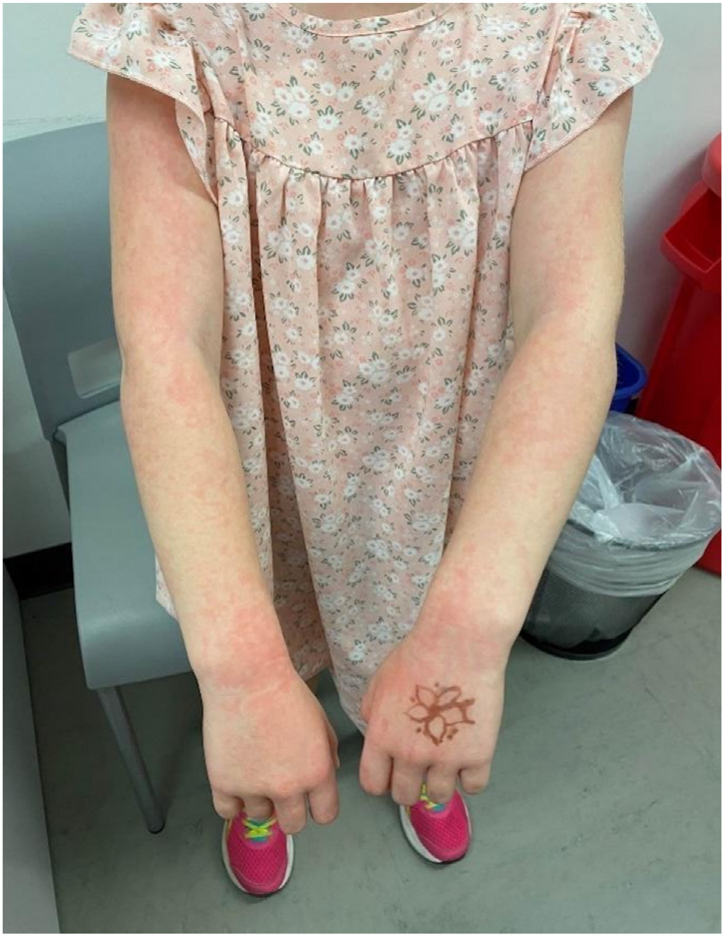
Image provided for teledermatology consultation demonstrating lace-like erythematous rash along the upper extremities of the patient.

**Fig 2:**
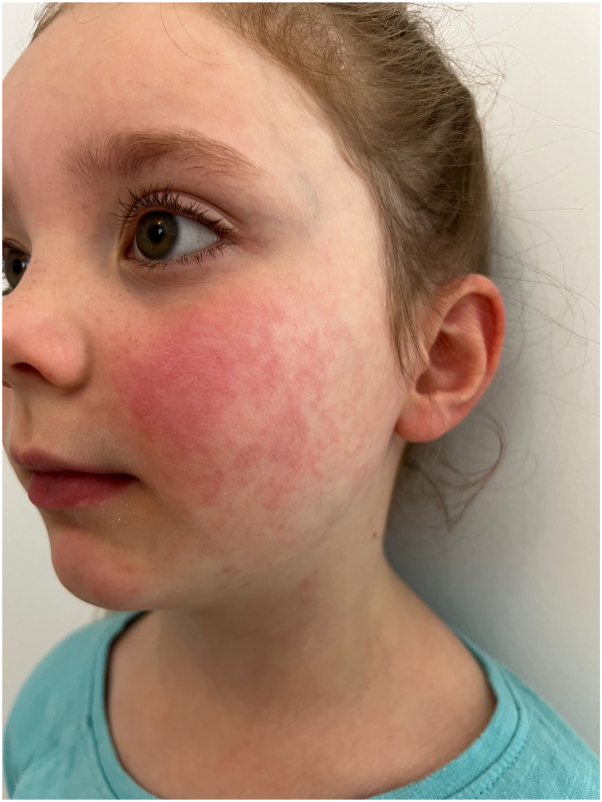
Image provided for teledermatology consultation demonstrating erythematous confluent rash in “slapped cheek” pattern.

An otherwise well child was sent to the school nurse after suddenly developing a net-like, pruritic rash on the arms and thighs associated with a bright red confluent rash on the cheeks. After calling the parents, a teledermatology consultation was sought for this dramatic rash to determine if it was infectious. The teledermatology reader report stated that the history of present illness included a 7-year-old child presented to the school nurse with sudden lace-like erythema on the bilateral arms and profoundly erythematous cheeks that suggested the patient was slapped. Prodrome: none. Prior treatment: none. Primary symptom: pruritus. Two images were provided with this teledermatology consultation. They demonstrated a lace-like erythematous rash along the lateral and dorsal aspects of the child’s arms, which extended onto the dorsum of the hands (Fig 1). A brightly erythematous confluent rash was noted on the cheeks (Fig 2). Based on the report, the consulted dermatologist determined that this viral exanthem is benign and self-limiting. Images showed that findings are pathognomonic for EI (“Fifth disease”). Reassurance and symptomatic treatment with over-the-counter pramoxine-containing moisturizing lotion, mild potency topical corticosteroid (hydrocortisone 2.5% cream), and an oral antihistamine (hydroxyzine 25 mg by mouth every night at bedtime) was recommended as needed for pruritus. Although this exanthem is not infectious at the time of the eruption, the child was sent home so that she could be aggressively treated for her pruritus with frequent topical applications of the prescribed medications.

## Discussion

Although there are limitations to teledermatology because of lighting, focus, composition, and bias to which lesions have been photographed,^7^ which can mask important clues, this case showcases how store-and-forward teledermatology was used to allow for important recommendations to be made to the school nurse. Research has indicated that accuracy in diagnosis and effectiveness in management have been comparable to in-person dermatological care, especially in regard to skin cancers.^7^, ^8^, ^9^ Pathognomonic features were visible in this EI case, demonstrating the potential usefulness of store-and-forward teledermatology in diagnosing inflammatory skin conditions as well. In other less clear-cut cases, research shows that teledermatology is beneficial for triage to either provide feedback for management to the referring clinician or recommending further in-person evaluation.^7^^,^^8^ Although there are studies that claim the clinical usefulness of store-and-forward teledermatology is limited,^10^ this case supports the functionality of teledermatology for diagnosis of distinct inflammatory conditions.

## Conflicts of interest

Dr Brodell has participated in multicenter clinical trials with CorEvitas (formerly Corrona) Psoriasis Registry, Sanofi, and Novartis and holds stock in Veradermics, Inc. Author Bernat and Dr Dolohanty have no conflicts of interest to declare.
